# Decreased expression of H19/miR-675 ameliorates muscle atrophy by regulating the IGF1R/Akt/FoxO signaling pathway

**DOI:** 10.1186/s10020-023-00683-w

**Published:** 2023-06-21

**Authors:** He Zhang, Fei Wang, Xiangsheng Pang, Yue Zhou, Shiming Li, Wenjiong Li, Peng Zhang, Xiaoping Chen

**Affiliations:** 1 National Key Laboratory of Human Factors Engineering, China Astronaut Research and Training Center, Beijing, China; 2 National Key Laboratory of Space Medicine, China Astronaut Research and Training Center, Beijing, China; 3 Department of Physical Education, Central South University, Changsha, Hunan China; 4 School of Sport Science, Beijing Sport University, Beijing, China

**Keywords:** LncRNA H19, miR-675, Muscle atrophy, Hindlimb suspension, Starvation, IGF1R

## Abstract

**Background:**

Long non-coding RNA (lncRNA) H19 is one of the most highly expressed and conserved transcripts in mammalian development, and its functions have been fully discussed in many contexts including tumorigenesis and skeletal muscle development. However, its exact role in muscle atrophy remains largely unknown. This study investigated the effect of lncRNA H19 on muscle atrophy and the potential underlying mechanism.

**Methods:**

Hindlimb suspension (HS) of C57BL/6 mice and starvation of C2C12 cells with PBS were conducted to induce atrophy. Real-time PCR and Western blotting were used to measure the expression of RNAs and proteins. LncRNA H19 and its encoded miR-675 were overexpressed or inhibited in different models of muscle atrophy. Immunofluorescence was carried out to examine the cross-sectional area (CSA) and minimal Feret’s diameter (MFD) of myofibers and myotube diameter.

**Results:**

The expression levels of lncRNA H19 and miR-675 were significantly reduced in both the soleus and gastrocnemius muscles in response to HS. Overexpression of lncRNA H19 led to an increase in *Atrogin-1* mRNA expression, and this effect was reversed by inhibiting miR-675. The overexpression of miR-675 aggravated both HS- and starving-induced muscle atrophy by inhibiting the IGF1R/Akt signaling pathway and promoting FoxO/Atrogin-1 expression. Conversely, miR-675 inhibition had the opposite effects.

**Conclusion:**

The lncRNA H19/miR-675 axis can induce muscle atrophy, and its downregulation in mice with HS-induced muscle atrophy may act as a protective mechanism against this condition.

**Supplementary Information:**

The online version contains supplementary material available at 10.1186/s10020-023-00683-w.

## Background

Skeletal muscle atrophy is a complex process that occurs in a variety of physiological and pathological conditions (e.g., limb immobilization, extended bed rest, sepsis, cancer cachexia, diabetes, congestive heart failure, and aging) that compromises quality of life and increases morbidity and mortality (Cao et al. 2018). This condition occurs when the balance between protein anabolic and catabolic processes are shifted in favor of net protein degradation. Reductions in protein synthesis or increases in protein degradation cause muscle atrophy. It has been reported that the ubiquitin–proteasome pathway accounts for most protein degradation in mammalian muscle atrophy (Khalil 2018). In the process of ubiquitination, E3 ubiquitin ligases play an important role in determining which proteins are degraded and in controlling the proteolysis rate (Sandri 2013). Two specific E3 ubiquitin ligases, muscle ring finger1 (MuRF1) and muscle atrophy F-box (MAFbx, also known as Atrogin-1) are upregulated and involved in various models of muscle atrophy (Bodine et al. 2001). For example, nine days of dexamethasone treatment resulted in more than tenfold increase in expression of both *Atrogin-1* and *MuRF1* genes in gastrocnemius muscle of rats (Bodine et al. 2001). Knockout of *Atrogin-1* or *MuRF1* genes exhibited 56% and 36% sparing of muscle mass respectively as compared with the wild type mice after 14 days of denervation (Bodine et al. 2001). These two genes are therefore called atrogenes and are considered markers of skeletal muscle atrophy. Nevertheless, additional E3 ubiquitin ligases may also contribute to muscle atrophy. MuRF1, for example, cooperates with MuRF2 to inhibit de novo protein translation (Witt et al. 2008).

Long noncoding RNAs (lncRNAs), which includes all RNA transcripts greater than 200 bp in length with little or no coding potential, are involved in numerous important biological processes (Guttman and Rinn 2012). They are found in both the nucleus and the cytoplasm, can be divided into multiple functional categories, and act on every aspect of gene expression (Batista and Chang 2013). In the nucleus, they activate or repress transcription by recruiting chromatin remodelers and modifiers to shape chromatin status (Chu et al. 2011). Some also influence RNA splicing by interacting with the RNA sequences (Liz et al. 2014) and still others act as protein/structural scaffolds (Yang et al. 2011). In the cytoplasm, lncRNAs show sequence complementarity with transcripts that originate from either the same chromosomal locus or independent loci. It is worth noting that one type of cytoplasmic lncRNA, namely, competing endogenous RNA (ceRNA), can regulate both the translation and the degradation rates of mRNAs by serving as a molecular sponge for miRNAs, thereby leading to a reduction in unbound miRNAs available as mRNA targets (Cesana et al. 2011).

LncRNA H19 is encoded by the *H19* gene, which is highly expressed in developing embryos and adult skeletal muscle (Gabory et al. 2010). LncRNA H19 was first described as an oncofetal transcript (Ghafouri-Fard et al. 2020). It can function as a primary microRNA precursor and its exon 1 encodes two conserved microRNAs, miR-675-3p and miR-675-5p (Cai and Cullen 2007). Parts of lncRNA H19’s function is mediated through miR-675 (Luo et al. 2019; Wang et al. 2020; Li et al. 2016). Numerous studies have reported the functions of lncRNA H19 and miR-675 in the pathogenesis of human cancers, including gastric cancer (Yan et al. 2017; Chen et al. 2022) and cholangiocarcinoma (Müller et al. 2019). However, it is also involved in skeletal muscle development, differentiation, and regeneration, as well as the pathogenesis of muscular dystrophy (Zhang et al. 2020). Mice carrying a target deletion of the *H19* gene show muscle hypertrophy as evidenced by a general increase in muscle mass (Martinet et al. 2016). However, the exact role of lncRNA H19 in muscle atrophy remains largely unknown.

In this study, we investigated the expression of lncRNA H19/miR-675 in mice with HS-induced muscle atrophy. By overexpressing or inhibiting miR-675 in vivo and in vitro, we confirmed the essential role of miR-675 in mediating muscle atrophy by regulating the IGF1R/Akt/FoxO pathway, as well as its potential as a new therapeutic target for combating skeletal muscle atrophy.

## Materials and methods

### Animals

Adult C57BL/6 mice (male, 8-week-old, 20 ± 2 g) were raised at room temperature under 12 h light and 12 h dark with free access to standard maintenance diet and water. All mice were purchased from Vital River Laboratories (Beijing, China) and the animal procedures were conducted in accordance with standard ethical guidelines and approved by the Institutional Animal Care and Use Committee China Astronaut Research and Training Center (ACC-IACUC-2022-018).

### Hindlimb suspension

To induce HS, animals were elevated sufficiently to prevent their hindlimbs from touching the cage floor or sides as described before (Wang et al. 2017a). All mice could reach food and water freely using their forelimbs. Control mice were raised normally until being euthanized. The mice with comparable body weight were randomly divided into two groups: the HS group underwent 7 days of HS and the control group was raised for 7 days under normal conditions (n = 12 in each group). All mice were sacrificed at the end of treatment by cervical dislocation. The soleus and gastrocnemius muscles were immediately removed, weighed, and frozen in liquid nitrogen.

### Cell culture and starvation

C2C12 cells (1101MOU-PUMC000099) were purchased from China Infrastructure of Cell Line Resource and were cultured in growth medium (GM) consisting of DMEM (Gibco, Waltham, MA, USA) supplemented with 10% (v/v) fetal bovine serum (Gibco, Waltham, MA, USA) and 1% penicillin/streptomycin (Hyclone, UT, USA) at 37℃ and 5% CO_2_. Cells grown to 80% confluency were switched into differentiation medium (DM) consisting of DMEM supplemented with 2% horse serum (Gibco, Waltham, MA, USA) and 1% penicillin/streptomycin to form myotubes. The in vitro atrophy model was induced by myotube starvation. Mature myotubes were treated with PBS containing Ca^2+^ and Mg^2+^ instead of DM for 4 h before harvest (Wang et al. 2017b). Three biological replicates with three technical replicates in each repetition were carried out in all in vitro experiment.

### Plasmid construction

To make plasmids of H19 that express full-length human H19 (pH19), PCR was carried out using human full-term placental cDNA as a template. The following sequences were used (where the lowercase letters are restriction enzyme sites for NotI and BamHI for the forward and reverse primers, respectively, and the uppercase letters are complementary to H19):

5′-aaggaaaaaagcggccgcAGCAGGGTGAGGGAGGGGGTG (forward).and 5′-cgcggatccGTAACAGTGTTTATTGATGATGAGTC (reverse). The resulting 2663 bp long PCR fragment was ligated to pcdna3.1 opened with NotI and BamHI.

### Transfection of plasmid, siRNA, and mature microRNAs

On the fifth day of C2C12 cells differentiation, H19 plasmid (8 μg) and H19 smart silencer (100 nM) were transfected into myotubes to regulate the RNA level of H19. Mimic (100 nM) and inhibitor (100 nM) of miR-675, and the same concentrations of corresponding negative controls, were used to regulate the RNA level of miR-675. Lipofectamine 2000 Reagent (Invitrogen, CA, USA) was used to conduct all transfections following the manufacturer’s instructions. Then the cells were treated with PBS for 4 h at 24 h after transfection. Cells were harvested for RNA, protein detection, and immunofluorescence assays after starvation.

LncRNA H19 smart silencer, miR-675 mimic, and miR-675 inhibitor, along with the corresponding negative controls, were purchased from RiboBio, China. The following sequences were used: mmu-miR-675-3p, CUGUAUGCCCUAACCGCUCAGU; mmu-miR-675-5p, UGGUGCGGAAAGGGCCCACAGU. The sequences for the H19 smart silencer are listed in Additional file 1: Table S1.

### Administration of agomiRs or antagomiRs

Twelve 8-week-old C57BL/6 J mice were anesthetized with 1% pentobarbital sodium (50 mg/kg), and their unilateral hindlimbs of each mouse were shaved and disinfected with 75% alcohol. AgomiRs or antagomiRs of miR-675-3p and their corresponding negative controls were injected into unilateral gastrocnemius muscles (two injection sites were selected from the medial and lateral muscles) before and at the fourth day of HS. The volume for each injection site was 10uL at the dose of 1 nmol. After HS, gastrocnemius muscles were harvested for RNA, protein, and immunofluorescence assays.

### RNA isolation and real-time PCR

Trizol reagent (Invitrogen, CA, USA) was used to extract total RNA from cells and tissue samples according to the manufacturer’s protocol (Invitrogen, 15596-026). cDNA synthesis for mRNA detection was carried out using the PrimeScript RT reagent kit (Takara RR037A, Japan). MicroRNA was reverse transcribed using the miScript® II RT Kit (QIAGEN, China). Real-time PCR was carried out using Step-One Plus with Power SYBR® Green (Applied Biosystems, USA). Data were analyzed using the comparative Ct method (2^−∆∆CT^) and normalized to GAPDH or U6. The sequences of the primers are listed in Additional file 1: Table S1.

### Western blot assay

Muscle tissues or cells were lysed in RIPA buffer (50 nM Tris–HCL, pH 7.4, 150 nM NaCl, 1% NP-40, 0.1% SDS) on ice for 30 min. The supernatant was centrifuged for 10 min at 12,000 g and 4 °C and qualified using the BCA protein assay kit (Thermo Scientific, Waltham, MA, USA). Proteins were separated via SDS-PAGE and then transferred to nitrocellulose membranes. Primary antibodies against IGF-1R (CST #9750), pAKT (CST #4060), FoxO3a (CST #12829) (Cell Signaling Technology), Atrogin-1 (ECM Biosciences, AP2041), MuRF1 (ECM Biosciences, MP3401), and GAPDH (MBL, Japan) were diluted 1:1000 in 5% skim milk overnight at 4℃. Then the membranes were incubated with secondary antibodies (anti-rabbit IgG or anti-mouse IgG, 1:1000 dilution, MBL, Japan) conjugated with horse radish peroxidase (HRP) for 2 h at room temperature. Protein bands were visualized using an enhanced chemiluminescence assay (ECL, Thermo Scientific, Waltham, MA, USA). The background was subtracted, and the signal of each target band was normalized to GAPDH.

### Immunofluorescence and histological analysis

The histological analysis was performed as described previously (Zhang et al. 2019). The cross-sectional area and minimal Feret’s diameter of the gastrocnemius muscles were determined by immunostaining with an anti-laminin antibody (dilution: 1:200; Abcam, Cambridge, MA, USA) (Moresi et al. 2010). The primary antibody was detected by Alexa Fluor-594 (dilution: 1:1000; Life Technologies, Carlsbad, CA, USA) fluorescent dye conjugated to an anti-rabbit secondary antibody. Myotubes were fixed with 4% paraformaldehyde for 10 min, blocked with 5% goat serum for 30 min, and incubated with anti-myosin (dilution: 1:500; ZSGB-BIO, China) at 4 °C overnight. The secondary antibody was goat anti-mouse IgG H&L (Alexa Fluor® 488) (dilution: 1:1000; Life Technologies, Carlsbad, CA, USA). Nuclei were stained using Vectashield with DAPI (Vector, USA). Images were visualized through a Leica TCS SPS III confocal microscope (Leica, Germany). The diameter of each individual myotube was measured and the average overall diameter was calculated. Images were analyzed using Image-Pro Plus 6.0.

### Statistical analysis

All data were analyzed using SPSS 23.0 software, and are presented as mean ± standard deviation. The Independent Samples *t*-test or Welch’s *t*-test were used to determine significance depending on the homogeneity of variance when two groups were compared. Comparisons of data among multiple groups were conducted via one-way ANOVA test followed by Tukey post doc analysis. *P* < 0.05 was considered a significant difference.

## Results

### H19/miR-675 is reduced during muscle atrophy

**Fig. 1:**
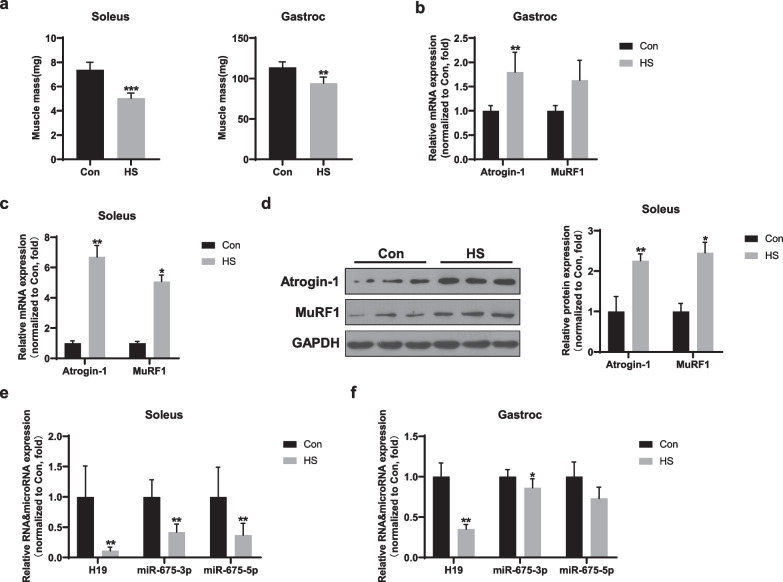
H19/miR-675 was suppressed in mice with HS-induced muscle atrophy. **a** Changes in mass of the soleus and gastrocnemius muscles after 7 days of HS (n = 6). **b**, **c** Real-time PCR analysis of the expression of *Atrogin-1* and *MuRF1* mRNA in the gastrocnemius and soleus muscles after HS (n = 6). **d** Western blotting analysis of the expression of Atrogin-1 and MuRF1 protein in the soleus muscles after HS (n = 6). Left panel: representative Western blotting images. Right panel: relative quantitative expression. **e, f** Real-time PCR analysis of the expression of H19, miR-675-3p, and miR-675-5p RNA in the soleus and gastrocnemius muscles after HS (n = 6). All data are shown as mean ± SD. *P < 0.05 and **P < 0.01 compared to the control group

Seven days of HS was sufficient to induce hindlimb muscle atrophy in mice, with the significant loss observed in soleus and gastrocnemius muscles (Fig. 1a). As expected, *Atrogin-1* and *MuRF1*, the two most well-known atrophy-related genes, were upregulated in gastrocnemius muscles after HS (Fig. 1b). The mRNA levels of *Atrogin-1* and *MuRF1* were also upregulated in soleus muscles as well as their protein expressions (Fig. 1c, d). However, H19, which is upregulated in several skeletal muscle atrophy models, was downregulated after HS in soleus (Fig. 1e). Meanwhile, its encoded microRNAs, miR-675-3p and miR-675-5p, were also reduced in atrophied muscles (Fig. 1e). Interestingly, the expression of H19/miR-675 showed similar patterns in gastrocnemius muscle after HS but to a less extent (Fig. 1f). These data indicate that the expression levels of H19/miR-675 may be closely associated with the progress of muscle atrophy.

### miR-675 mediates the effect of H19 on Atrogin-1 expression

**Fig. 2:**
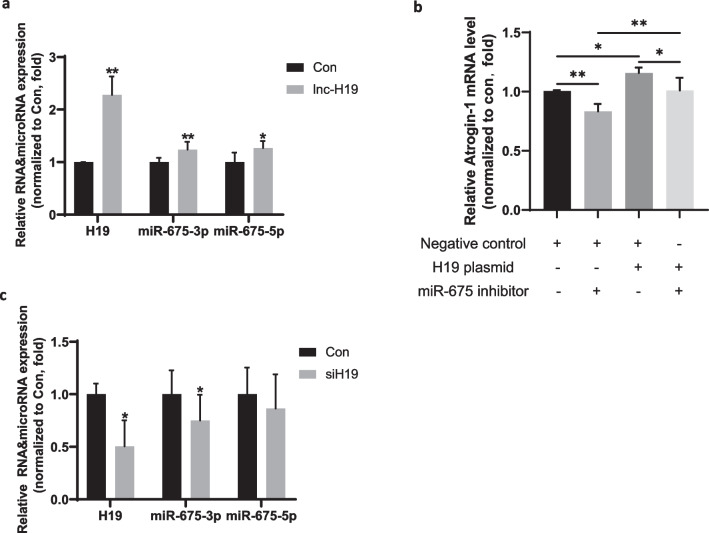
Effect of H19/miR-675 on Atrogin-1 expression in vitro. **a** Real-time PCR analysis of the expression of H19, miR-675-3p, and miR-675-5p in C2C12 cells treated with H19 overexpression plasmid (n = 3). **b** Real-time PCR analysis of the expression of *Atrogin-1* mRNA in C2C12 cells transfected with H19 overexpression plasmid either alone or in combination with miR-675 inhibitors (n = 3). **c** Real-time PCR analysis of the expression of H19, miR-675-3p, and miR-675-5p in C2C12 cells treated with H19 smart silencer (n = 3). All data are shown as mean ± SD. *P < 0.05 and **P < 0.01 compared to control groups

Next, to demonstrate the involvement of H19/miR-675 in muscle atrophy, we measured the effect of H19/miR-675 on the expression of Atrogin-1 and MuRF1. H19 overexpression plasmid or smart silencer was transfected into C2C12 cells to increase or decrease H19 expression, respectively. RT-qPCR confirmed that H19 expression was increased in muscle cells transfected with H19 overexpression plasmid; miR-675-3p and miR-675-5p were also upregulated, as expected (Fig. 2a). H19 overexpression had no effect on *MuRF1* expression (Additional file 1: Fig. S1a) but increased the mRNA expression of A*trogin-1*(Fig. 2b). When cells were co-transfected with H19 overexpression plasmid and miR-675 inhibitor, the increased mRNA expression of *Atrogin-1* induced by H19 was completely blocked (Fig. 2b). miR-675 inhibition was also sufficient to decrease the expression of *Atrogin-1* (Fig. 2b). H19 expression was decreased in cells transfected with H19 smart silencer, which resulted in the downregulation of miR-675-3p but not miR-675-5p (Fig. 2c). However, this did not significantly reduce the expression of *Atrogin-1* (Additional file 1: Fig. S1b). These results indicate that changes in H19/miR-675 expression, specifically a decrease in the expression of miR-675, may act as a compensatory mechanism in response to an atrophic stimulus.

### Inhibition of miR-675 attenuates muscle atrophy in vivo and in vitro

**Fig. 3:**
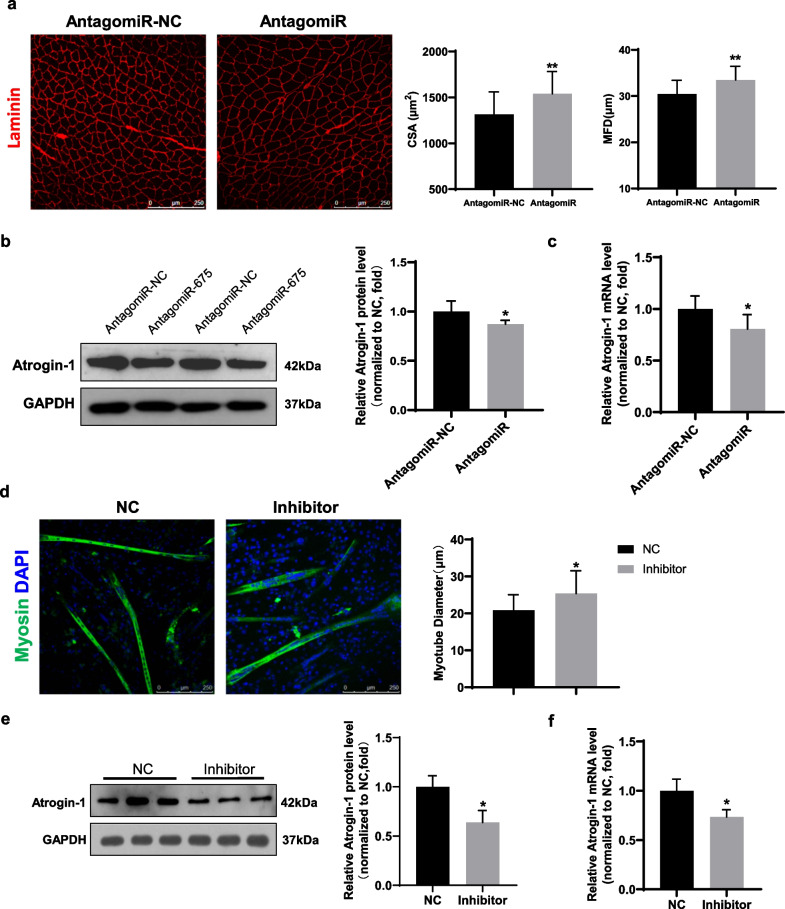
Inhibition of miR-675 ameliorated muscle atrophy in vivo and in vitro. **a** Representative immunofluorescence images of the gastrocnemius muscles transfected with miR-675 antagomiR or negative control (n = 6). Left bar panel: cross-sectional area (CSA). Right bar panel: minimal Feret’s diameter (MFD). The anti-laminin stained sections were used for measurement of size of myofibers. Scale bar: 250 μm. **b** Western blotting and **c** real-time PCR analyses of Atrogin-1 expression in muscles transfected with either miR-675 antagomiR or negative control (n = 6). **d** Representative immunofluorescence images of myotubes in C2C12 cells (n = 3). Myotubes were stained with anti-myosin antibody (green). Nuclei were stained with DAPI (blue). Scale bar: 250 μm. **e** Western blotting and **f** real-time PCR analyses of Atrogin-1 expression in C2C12 myotubes transfected with either miR-675 inhibitor or negative control (n = 3). The CSA, MFD, the diameters of myotubes, and the intensities of protein bands were quantified using Image-J software. All data are shown as mean ± SD. *P < 0.05 and **P < 0.01 compared to the negative control (NC) groups

Next, we tested whether inhibition of miR-675 would attenuate muscle atrophy (Fig. 3). miR-675 antagomiR was transfected into gastrocnemius muscle to repress the function of miR-675, while negative control was transfected into the contralateral gastrocnemius muscle. The antagomiR led to greater myofiber size in HS mice, indicating the inhibition of muscle atrophy (Fig. 3a). Meanwhile, HS-induced Atrogin-1 expression was suppressed by miR-675 antagomiR at both the protein and mRNA levels (Fig. 3b, c). Furthermore, we validated the effect of miR-675 on other types of muscle atrophy. C2C12 myotubes were transfected with miR-675 inhibitors and then was induced to muscle atrophy by starving. Inhibition of miR-675 led to an increase in myotube size (Fig. 3d) and a decrease in Atrogin-1 expression (Fig. 3e, f). Taken together, these data suggest that reduced expression of miR-675 may be an adaptive response against muscle atrophy.

### miR-675 overexpression aggravates muscle atrophy in vivo and in vitro

**Fig. 4:**
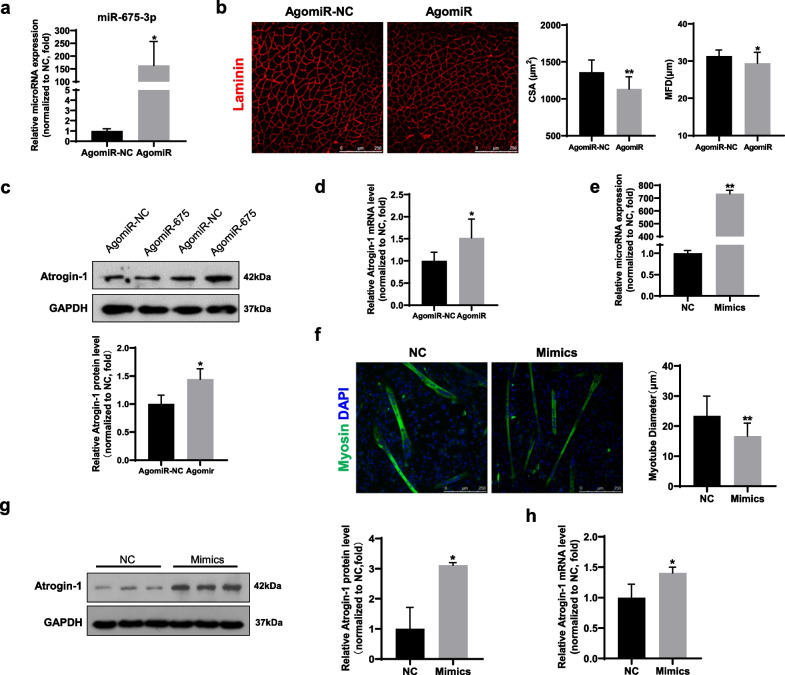
Overexpression of miR-675 exacerbated muscle atrophy in vivo and in vitro. **a** Real-time PCR analysis of miR-675-3p expression in gastrocnemius muscles transfected with either miR-675 agomiR or negative control (n = 6). **b** Representative immunofluorescence images of gastrocnemius muscles transfected with miR-675 agomiR or negative control (n = 6). Left bar panel: cross-sectional area (CSA). Right bar panel: minimal Feret’s diameter (MFD). The anti-laminin stained sections were used for measurement of size of myofibers. Scale bar: 250 μm. **c** Western blotting and **d** real-time PCR analyses of Atrogin-1 expression in muscles transfected with miR-675 agomiR or negative control (n = 6). **e** Real-time PCR analysis of miR-675-3p in C2C12 myotubes transfected with miR-675 mimics or negative control (n = 3). **f** Representative immunofluorescence images of myotubes transfected with miR-675 mimics or negative control (n = 3). Myotubes were stained with anti-myosin antibody (green). Nuclei were stained with DAPI (blue). Scale bar: 250 μm. **g** Western blotting and **h** real-time PCR analyses of Atrogin-1 expression in C2C12 myotubes transfected with miR-675 mimics or negative control (n = 3). The CSA, MFD, the diameters of myotubes, and the intensities of protein bands were quantified using Image-J software. All data are shown as mean ± SD. *P < 0.05 and **P < 0.01 compared to the control groups

To further confirm the effect of miR-675 on muscle atrophy, we transfected miR-675-3p agomiR into gastrocnemius muscles to increase the expression of miR-675-3p; the contralateral gastrocnemius muscle was transfected with negative control agomiR (Fig. 4). After 7 days of HS, the RNA level of miR-675-3p was significantly upregulated in muscle injected with miR-675-3p agomiR compared to contralateral muscles (Fig. 4a). Meanwhile, miR-675-3p overexpression lowered the myofiber size (Fig. 4b) and enhanced the expression of Atrogin-1 more than that of contralateral muscle (Fig. 4c, d). Similarly, miR-675 mimic was used to increase miR-675 expression in C2C12 myotubes before it was starved in PBS for 4 h (Fig. 4e). In agreement with the in vivo results, miR-675 overexpression aggravated starved-induced myotube atrophy (Fig. 4f), accompanied by increased expression of Atrogin-1 (Fig. 4g, h). These data demonstrate that miR-675 overexpression exacerbates muscle atrophy induced by multiple conditions.

### miR-675 mediates muscle atrophy by targeting the IGF1R/Akt/FoxO signaling pathway

**Fig. 5:**
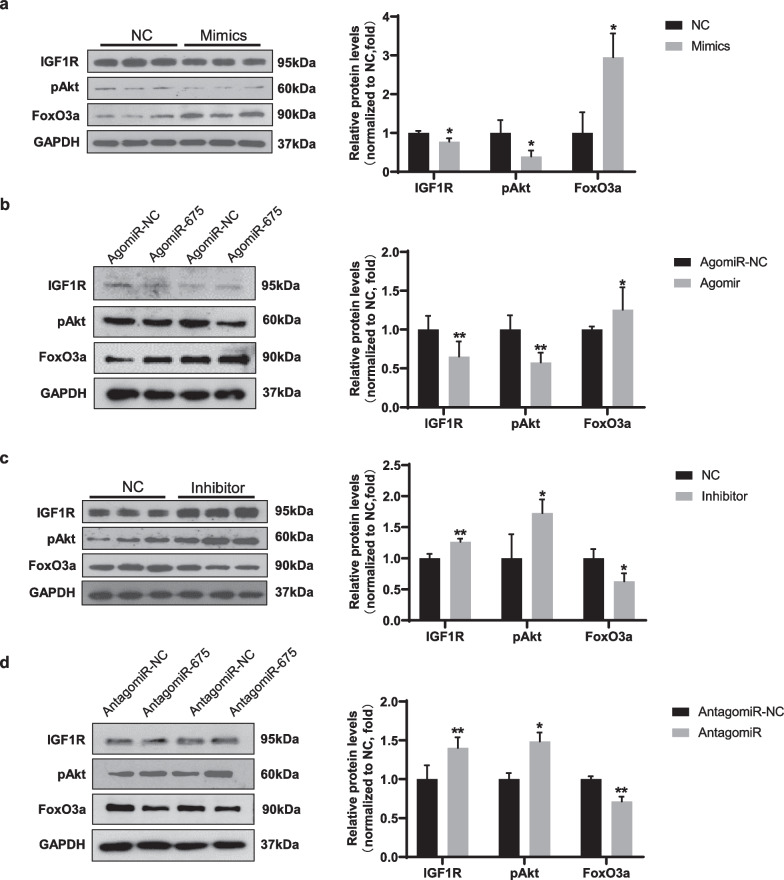
Effect of miR-675 on the IGF1R/Akt/FoxO signaling pathway during muscle atrophy. Western blotting analysis of IGF1R, pAKT, and FoxO3a in C2C12 myotubes transfected with miR-675 mimics **a** or inhibitor **c** and their negative controls (n = 3). Western blotting analysis of IGF1R, pAKT, and FoxO3a in gastrocnemius muscles transfected with miR-675 agomiR **b** or antagomiR **d** and their negative controls (n = 6). All data are shown as mean ± SD. *P < 0.05 and **P < 0.01 compared to the control groups

IGF1R is a target gene of miR-675. To investigate whether miR-675 promotes muscle atrophy by targeting IGF1R, we investigated the effect of miR-675 on IGF1R expression after muscle atrophy (Fig. 5). Overexpression of miR-675 resulted in decreased expression of IGF1R and its downstream kinase pAKT in both in vitro (Fig. 5a) and in vivo (Fig. 5b) muscle atrophy models. Accordingly, FoxO3a, the well-known downstream target of Akt and the main transcriptional factor regulating Atrogin-1 expression, was upregulated by overexpression of miR-675 in atrophied cells (Fig. 5a) and muscles (Fig. 5b). Conversely, inhibition of miR-675 increased the expression of IGF1R and pAKT but decreased that of FoxO3a in both muscle atrophy models (in vitro: Fig. 5c, in vivo: Fig. 5d). These results indicate that miR-675 mediates muscle atrophy by targeting the IGF1R /Akt/FoxO signaling pathway.

## Discussion

Muscle atrophy is a complication of many diseases that leads to weakness and metabolic comorbidities with a high societal and financial cost (Ding et al. 2018). Over the past several decades, much progress has been made studying the mechanism of muscle atrophy, especially the intracellular signaling mechanisms involved (Cid-Díaz et al. 2021; Silva et al. 2015; Waning et al. 2015). However, the condition needs to be further explored.

LncRNA H19 is a well-characterized lncRNA that is highly expressed in embryonic tissues but downregulated after birth in all tissues except skeletal muscle. It plays essential roles in muscle growth and development (Dey et al. 2014; Geng et al. 2018; Xu et al. 2017) and may be associated with low muscle mass. For instance, lncRNA H19 is significantly upregulated in denervation-induced muscle atrophy, the late phrase of amyotrophic lateral sclerosis (Hitachi et al. 2020; Alessio et al. 2019), as well as in COPD patients with a low fat-free mass index and low muscle strength (Lewis et al. 2016). However, we found that lncRNA H19 is decreased rather than increased in HS-induced muscle atrophy. The microRNAs that it encodes, miR-675-3p and miR-675-5p, were also downregulated in atrophied muscle induced by HS. Our findings agree with a previous study that reported a slight decrease in lncRNA H19 expression in tibialis anterior muscles (TA) of mice exposed to HS and a significantly reduced expression of lncRNA H19 in TA of mice after fasting (Hitachi et al. 2020). These distinct expression patterns of H19 in various atrophy models are largely due to different atrophic stimuli. Another interesting observation unveiled in this study is the disparity of H19/ miR-675 expression in the gastrocnemius *versus* the soleus. It is well-known that slow-twitch soleus muscle loses more muscle mass than that of gastrocnemius during muscle wasting (Ciciliot et al. 2013), as was also shown in this study. The mRNA level of both *Atrogin-1* and *MuRF1* were upregulated with over fivefold change in soleus *versus* 1.5-fold change in gastrocnemius muscle. Notably, lncRNA H19 and its encoded miR-675 were reduced to a greater extent in soleus muscle than in gastrocnemius muscle, suggesting that reduced H19/miR-675 expression is closely associated with loss of muscle mass.

Although the correlation between altered expression of lncRNA H19 and the loss of muscle mass has also been previously reported (Martinet et al. 2016), its exact role has remained largely unknown. It was reported that mice with *H19* deficiency display an overgrowth phenotype with muscle hypertrophy and hyperplasia compared to wild-type littermates (Martinet et al. 2016). It may also act as a negative regulator for cardiomyocyte hypertrophy (Liu et al. 2016). Such studies suggest that lncRNA H19 may contribute to muscle atrophy. In this study, we demonstrated for the first time that overexpression of lncRNA H19 in vitro was sufficient to upregulate *Atrogin-1* expression. Accumulating evidence indicates that miR-675 mediates most effects of lncRNA H19 on myogenic differentiation and slows the proliferation of different types of cells (Keniry et al. 2012). In our study, the effect of lncRNA H19 on *Atrogin-1* mRNA expression was completely reversed by co-transfection with miR-675 inhibitor, indicating that the atrophic effect of lncRNA H19 is also attributed to miR-675. Although knockdown of H19 in C2C12 cells only caused a slight decrease in the expression of *Atrogin-1*, inhibition of miR-675 downregulated *Atrogin-1* expression. We thus speculate that changes in lncRNA H19/miR-675, specifically decreased miR-675 expression, may act as a protective mechanism against muscle atrophy. This hypothesis was confirmed by in vivo and in vitro experiments. We found that miR-675 overexpression upregulated the expression of Atrogin-1, thereby resulted in smaller myofibers in mice subjected to HS or smaller myotubes upon fasting, indicating aggravated muscle atrophy. In contrast, inhibition of miR-675 protected against both in vivo and in vitro muscle atrophy by downregulating the expression of Atrogin-1. Collectively, our results reveal the essential role of miR-675 in inducing muscle atrophy and that decreased miR-675 expression acts as an adaptive mechanism against muscle atrophy.

IGF1R belongs to the receptor tyrosine kinase family and is composed of a membrane-spanning heterotetramer (Vassilakos and Barton 2018). The binding of IGF1 to IGF1R induces its autophosphorylation and creates a docking site for the recruitment and/or phosphorylation of substrates that lead to the activation of the phosphoinositide 3-kinanse (PI3K)/Akt serine/threonine kinase signaling pathway. Activated Akt phosphorylates multiple substrates including the Forkhead box class O (FoxO) transcription factors, which negatively regulate muscle growth (Bonaldo and Sandri 2013). Phosphorylation of FoxO by Akt blocks nuclear translocation and thus prevents expression of these atrogenes, particularly Atrogin-1 and MuRF1 (Sandri et al. 2004). *IGF1R* is a predicted target of miR-675-3p and contains two 7-mer seed matches in its 3′-untranslated region (3′-UTR) (Keniry et al. 2012). Our results showed that overexpression of miR-675-3p caused a significant decrease in IGF1R/pAkt expression and an increase in FoxO3a and Atrogin-1 protein levels, while inhibition of exogenous miR-675-3p led to the opposite changes. Reduced expression of miR-675 may slow down the process of muscle atrophy by repressing the inhibition of AKT/FoxO3a by targeting IGF1R.

There are some limitations to this study. First, Smad1/5 are also target genes of miR-675-3p (Dey et al. 2014), and activation of the BMP/Smad1/5 pathway can induce muscle hypertrophy and inhibit atrophy (Winbanks et al. 2013). Therefore, whether miR-675-3p is involved in the process of muscle atrophy by targeting Smad1/5 remains to be further investigated. Second, the decrease in lncRNA H19 expression is closely related to insulin resistance. It is well known that disuse-induced muscle atrophy is accompanied by insulin resistance (Dirks et al. 2016). Therefore, it would be interesting to explore whether the decrease in lncRNA H19 expression contributes to the insulin resistance in that condition. Third, we observed a significant drop in the lncRNA H19 level after muscle atrophy, but the reason for this phenomenon remains unclear.

## Conclusions

This study revealed the expression of lncRNA H19/miR-675 during HS-induced muscle atrophy. We further defined the role of lncRNA H19/miR-675 in inducing muscle atrophy by targeting IGF1R, which provides clues for further elucidation of the mechanism of muscular atrophy and potential therapeutic targets.

## Supplementary Information

**Additional file 1: Figure S1.** **a** The expression of *MuRF1* mRNA in C2C12 cells transfected with H19 overexpression plasmid. **b** The expression of *Atrogin-1* mRNA in C2C12 cells transfected with H19 smart silencer. All data are shown as mean ± SD. **Table S1.** Primers and shRNA sequences used in this study.

## Data Availability

All data generated or analyzed during this study are included in this published article and its Additional files.

## Abbreviations

Akt: Protein kinase B
Atrogin-1: Muscle atrophy F-box
BMP: Bone morphogenetic protein
ceRNA: Competing endogenous RNA
CSA: Cross-sectional area
HS: Hindlimb suspension
FoxO: Forkhead box class O
IGF1R: Insulin growth factor 1 receptor
LncRNA: Long non-coding RNA
MuRF1: Muscle ring finger1
PI3K: Phosphatidylinositol3-kinase
Smad: Drosophila mothers against decapentaplegic protein
TA: Tibialis anterior muscles
