# Supplementary material for: Decreased expression of H19/miR-675 ameliorates muscle atrophy by regulating the IGF1R/Akt/FoxO signaling pathway
Source: Mol Med. 2023 Jun 21;29:78. doi: 10.1186/s10020-023-00683-w (PMC10286355; doi:10.1186/s10020-023-00683-w)
Supplement: Supplementary file 1 — Additional file 1: Figure S1. a The expression of MuRF1 mRNA in C2C12 cells transfected with H19 overexpression plasmid. b The expression of Atrogin-1 mRNA in C2C12 cells transfected with H19 smart silencer. All data are shown as mean ± SD. Table S1. Primers and shRNA sequences used in this study. [file 10020_2023_683_MOESM1_ESM.docx]

**Additional file 1**

Additional file 1: Table S1. Primers and shRNA sequences used in this study

| Genes (mmu) |  | Sequence |
| --- | --- | --- |
| H19 | forward | 5′-CCTCAAGATGAAAGAAATGGTGCTA-3′ |
|  | reverse | 5′-TCAGAACGAGACGGACTTAAAGAA-3′ |
| Atrogin-1 | forward | 5′-TCAGCAGCCTGAACTACGAC-3′ |
|  | reverse | 5′-GCGCTCCTTCGTACTTCCTT-3′ |
| MuRF1 | forward | 5′-GTGTGAGGTGCCTACTTGCT-3′ |
|  | reverse | 5′-GACTTTTCCAGCTGCTCCCT-3′ |
| MiR-675-3p |  | 5′-TGTATGCCCTAACCGCTCAGT-3′ |
| MiR-675-5p |  | 5′-TGGTGCGGAAAGGGCCCACAGT-3′ |
| Reverse |  | 5′-GAATCGAGCACCAGTTACGC-3′ |
| U6 | forward | 5′-GCTTCGGCAGCACATATACTAAAAT-3′ |
|  | reverse | 5′-CGCTTCACGAATTTGCGTGTCAT-3′ |
| GAPDH | forward | 5′-GAGAGTGTTTCCTCGTCCCG-3′ |
|  | reverse | 5′-ACTGTGCCGTTGAATTTGCC-3′ |
| H19 smart silencer |  | GCAGAATGGCACATAGAAA |
|  |  | GGATCCAGCAAGAACAGAA |
|  |  | GCAGTCATCCAGCCTTCTT |
|  |  | CTCCAGGGAGGTGATAGGAG |
|  |  | TCCGGTGTGATGGAGAGGAC |
|  |  | CCACCCACATCATCCTGGAG |

Additional file 1: Figure S1. (a) The expression of *MuRF1* mRNA in C2C12 cells transfected with H19 overexpression plasmid (n=3). (b) The expression of *Atrogin-1* mRNA in C2C12 cells transfected with H19 smart silencer (n=3). All data are shown as mean ± SD.
